# Mitochondrial DNA Variation, but Not Nuclear DNA, Sharply Divides Morphologically Identical Chameleons along an Ancient Geographic Barrier

**DOI:** 10.1371/journal.pone.0031372

**Published:** 2012-03-13

**Authors:** Dan Bar Yaacov, Karmit Arbel-Thau, Yael Zilka, Ofer Ovadia, Amos Bouskila, Dan Mishmar

**Affiliations:** Department of Life Sciences, Ben-Gurion University of the Negev, Beer-Sheva, Israel; University of Arkanas, United States of America

## Abstract

The Levant is an important migration bridge, harboring border-zones between Afrotropical and palearctic species. Accordingly, *Chameleo chameleon*, a common species throughout the Mediterranean basin, is morphologically divided in the southern Levant (Israel) into two subspecies, *Chamaeleo chamaeleon recticrista* (CCR) and *C. c. musae* (CCM). CCR mostly inhabits the Mediterranean climate (northern Israel), while CCM inhabits the sands of the north-western Negev Desert (southern Israel). AFLP analysis of 94 geographically well dispersed specimens indicated moderate genetic differentiation (PhiPT = 0.097), consistent with the classical division into the two subspecies, CCR and CCM. In contrast, sequence analysis of a 637 bp coding mitochondrial DNA (mtDNA) fragment revealed two distinct phylogenetic clusters which were not consistent with the morphological division: one mtDNA cluster consisted of CCR specimens collected in regions northern of the Jezreel Valley and another mtDNA cluster harboring specimens pertaining to both the CCR and CCM subspecies but collected southern of the Jezreel Valley. AMOVA indicated clear mtDNA differentiation between specimens collected northern and southern to the Jezreel Valley (PhiPT = 0.79), which was further supported by a very low coalescent-based estimate of effective migration rates. Whole chameleon mtDNA sequencing (∼17,400 bp) generated from 11 well dispersed geographic locations revealed 325 mutations sharply differentiating the two mtDNA clusters, suggesting a long allopatric history further supported by BEAST. This separation correlated temporally with the existence of an at least 1 million year old marine barrier at the Jezreel Valley exactly where the mtDNA clusters meet. We discuss possible involvement of gender-dependent life history differences in maintaining such mtDNA genetic differentiation and suggest that it reflects (ancient) local adaptation to mitochondrial-related traits.

## Introduction

Genetic variation within maternal lineages as reflected by mitochondrial DNA (mtDNA) is commonly used to trace ancient population migrations, demographic history, phylogeography and phylogeny. Since in vertebrates mtDNA population genetic analyses is confined to tracing the migration patterns of maternal lineages, a more complete picture of population genetic structure requires analysis of nuclear DNA (nDNA)-encoded markers inherited from both parents [1]. Moreover, being maternally inherited, the mtDNA population diversity likely reflects maternally-directed natal site fidelity, whereas genome-wide bi-parentally inherited nDNA markers assist in quantifying levels of gene flow among subpopulations for both sexes [1]. Notably, the maternal mode of inheritance of mtDNA in vertebrates confers a smaller effective population size compared to that of the nDNA. This could result in substantial differences in patterns of genetic diversity and population structure, especially when using both mtDNA and nDNA-encoded markers in the same study [2]. Indeed, several studies have demonstrated differences in patterns of Y chromosome and mtDNA variation in humans, probably reflecting differences between matriarchal or patriarchal life styles. For example, postmarital residence patterns in Native Americans have strongly influenced genetic structure, with patrilocal and matrilocal populations showing different patterns of male and female gene flow [3]. Similarly, the population genetic structure of various vertebrates [4], [5] could be influenced by differences in maternal and paternal migration patterns. In such cases, one sex specific marker would show even distribution of markers across geographic regions, whereas the variation pattern of a marker transmitted by the other sex would be characterized by local homogeneity and large inter-population differences (‘patchy’ distribution) [5]. Additionally, differences in the distribution of non-Y chromosome nDNA markers and mtDNA markers could be observed in hybrid zones [6]. Selection could act differently on mtDNA and nDNA-encoded genes, as well as influencing co-evolving mitochondrial and nuclear DNA-encoded factors [7], [8]. Thus selection could influence patterns of mtDNA and nDNA population variation differently [9]–[11]. Finally, such differences can also be used to show traces of genetic exchange between taxa in markers that pre-dated the emergence of phenotypic divergence [12]. In summary, differences in patterns of population genetic variability and structure between mtDNA and nDNA markers can be attributed to modes of inheritance and different spatial movement patterns of males and females. These patterns could also be affected by different actions of natural selection on the two genomes – a possibility that has yet to be assessed.

Israel, being at the southern Levant, is an important migration bridge between Africa and Eurasia, harboring border-zones between Afrotropical (formerly ‘Ethiopian’) and palearctic species. Chameleons in the Levant are divided into two subspecies based on morphology, *Chamaeleo chamaeleon recticrista* and *C. c. musae* (CCR and CCM, respectively). The first sub-species is found in northern Israel as well as in the northern part of the Levant (i.e. Turkey) and inhabits Mediterranean habitats (Coastal Plain, Judean Mountains, Galilee and the Golan Heights) whereas the second taxon is confined to the sandy habitats of southern Israel including the north-western Negev and the Sinai peninsula [13], [14]. Recently five whole mtDNA sequences of chameleons belonging to the same species (*C. chamaeleon*) were sampled from distant geographic locations throughout the near east and Mediterranean basin including one from Portugal [15], two from Turkey, one from Cyprus, and one from Yemen [16]. These sequences exhibited a correlation between geographic distance and the degree of sequence divergence (i.e., isolation by distance). However, no studies of genetic divergence of the known subspecies of *C. chamaeleon* in the Levant have been performed. Therefore we aimed at investigating the population structure of this species in the southern Levant (Israel).

Here, we investigated whether chameleon sub-species in the southern Levant corresponded with patterns of nuclear and/or mitochondrial markers, and whether these markers were congruent. To this end we collected chameleon samples throughout Israel, compared their morphological characteristics, and analyzed mtDNA and nuclear DNA (AFLP) variation.

## Results

**Figure 1:**
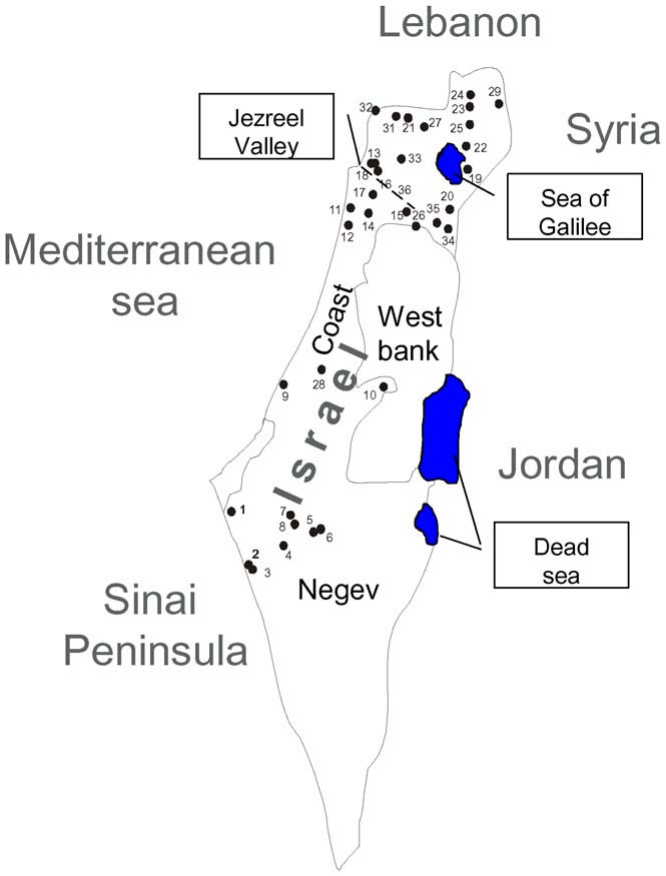
Map of chameleon collecting sites across Israel. 1.Avshalom, 2. Nitzana, 3. Kmehin, 4. Shivta Junction, 5. Seher Stream, 6. Ramat Beka, 7. Revivim, 8. Beer Aslug, 9. Nizzanim, 10. Jerusalem, 11. Habonim, 12. Ceasarea, 13. Kishon, 14. Tivon, 15. Salem, 16. Oosha, 17. Gahar mountain, 18. Carmel mountain, 19. Haon, 20. Kochav Hayarden, 21. Fasuta, 22. Ramot, 23. Akbara Stream, 24. Shamir, 25. Korazim, 26. Magen Shaul, 27. Baram, 28. Mazkeret Batia, 29. Avital mountain, 30. Banias, 31. Matat, 32. Rosh HaNikra, 33. Harrit, 34. Bait Shean, 35. Gilboa, 36. Jezreel Junction.

**Figure 2:**
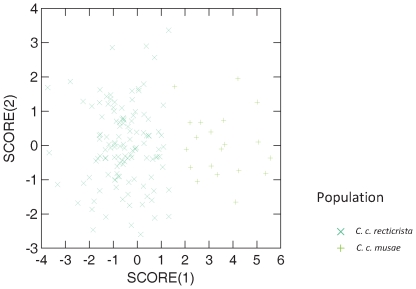
Results of a canonical discriminant function analysis of the morphological data set, demonstrating the separation between the desert (*C. c. musae*) and Mediterranean (*C. c. recticrista*) subspecies. The first canonical variate (score 1 = CV1), which provides the maximal separation among instars, is given by: CV1 = 0.345×logA×0.834×logB−0.013×logD−0.03×logE+0.257×logF+0.121×logG−0.91×logH+0.371×logI−0.45×logJ−1.49×logK+0.354×logL−1.043×logM−0.609×logN+0.83×logO (see Table S1, Fig. S1).

In order to assess patterns of morphological variation in chameleons in Israel, we performed multivariate analysis of variance (MANOVA) followed by a canonical discriminant function analysis (see Materials and Methods) of 14 morphological traits in a sample of 147 chameleons (Tables S1, S2). These chameleons were collected in 7 geographic regions, i.e., Nizzanim sand dunes in the southern Coastal Plain, the western Negev Desert, Jerusalem Mountains area, Carmel Mountain area, Haifa Bay area, Jezreel Valley, and several sites in northern Israel (including the Golan Heights, Upper and Lower Galilee, Kinnert area, Bet-Shean Valley and the Gilboa) (Fig. 1). The results of this analysis supported the former classification into two subspecies, *Chamaelo-chamaeleon recticristae* (CCR) and *Chamaelo-chamaeleon musae* (CCM) [13], as well as their geographical distribution in Israel (Fig. 2). Six morphological characters were sufficient to distinguish the CCR and CCM subspecies: crest–mouth length, crest length, arm length, medial foot pad length, medial hand pad length and eye diameter (Table S2). It is important to note that although the Nizzanim sand dunes appear to be a very similar habitat to the sands of the north-western Negev, all of the collected chameleons in Nizzanim sand dunes grouped within the CCR cluster rather than the CCM cluster. Using Principal Component Analysis (PCA), we found that PC1 and PC2 accounted for 78.84% and 8.64%, respectively, of the total variance between the CCR and CCM subspecies (Fig. 2, Fig. S1). All of the loadings of the components in PC1 were positive, and therefore this axis is interpreted as representing overall body size. Some of the PC2 loadings were positive and some negative so this axis was interpreted as representing variation in shape that was independent of body size. Even though the subspecies were separated on PC1, indicating a smaller body size of CCM compared to CCR, it was clear that the large morphological differentiation occurred along the PC2 axis. PCA performed only with head morphology or only the limb morphology showed no clear clustering, indicating that indeed it was the ratio between the head and the limb (shape) that distinguished the two subspecies. It is important to note that these size and shape differences between the two subspecies were not confounded by sexual dimorphism in size or shape (Fig. S1).

### Genomic DNA variation patterns further supports the morphology-based taxonomic division

**Figure 3:**
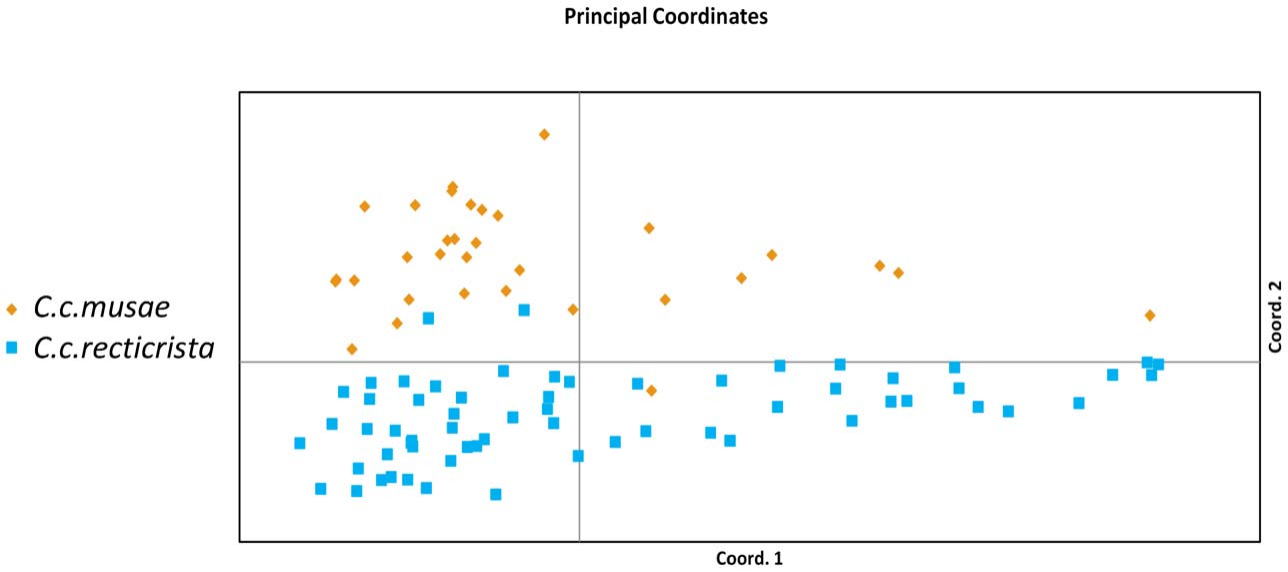
Principal coordinates analysis based on 323 AFLP loci detected in 94 chameleons sampled across different parts of Israel. Of the total genetic variation in the dataset, 54.3% was explained by the first two coordinates.

Principle coordinate analysis of the amplified fragment length polymorphism (AFLP) data, comprising of more than 300 different nDNA encoded loci in the 94 chameleon samples, revealed clear divergence of samples belonging to the two subspecies (Fig. 3). Two CCR individuals clustered with CCM (collected in sites 9 and 12; see Fig. 1), and one CCM individual clustered with CCR (collected in site 7 see Fig. 1). It is possible that these individuals represented traces of genetic mixture between CCM and CCR. However, because of the close proximity of human settlements to these sites, it is more likely that these specimens were simply transported by humans. AMOVA analyses indicated moderate genetic differentiation [17] between geographical regions separating the two subspecies (Table 1; PhiPT = 0.097, p<0.0001). Therefore, analysis of multiple nDNA-encoded loci revealed the existence of two clusters correlated with the morphological division of chameleons in Israel into two subspecies (i.e., CCR and CCM).

**Table 1:** Analysis of Molecular Variance (AMOVA) of AFLP results*.

Source	Df	SS	MS	Est. Var.	%
Among Populations	1	181.317	181.317	3.518	10%
Within Populations	92	3019.981	32.826	32.826	90%
Total	93	3201.298		36.344	100%

* General PhiTP value calculated from the 2 morphological sub species. PhiPT value is analog to Fst, where values ranging from 0.15–0.25 reflect great genetic differentiation, values ranging from 0.05–0.15 reflect moderate genetic differentiation and under 0.05 suggest little genetic differentiation. AMOVA were perform using Genalex 6.3 for excel 2007. Df – degrees of freedom; SS – sum of square, MS- mean square, Est. Var. – estimated variance.

Among populations – between the 2 morphological sub species (i.e. between CCR and CCM). Within populations – within each of the 2 morphological sub species (i.e. CCR and CCM).

### Mitochondrial DNA sequence analysis reveal notable population divergence unrelated to taxonomic division

**Figure 4:**
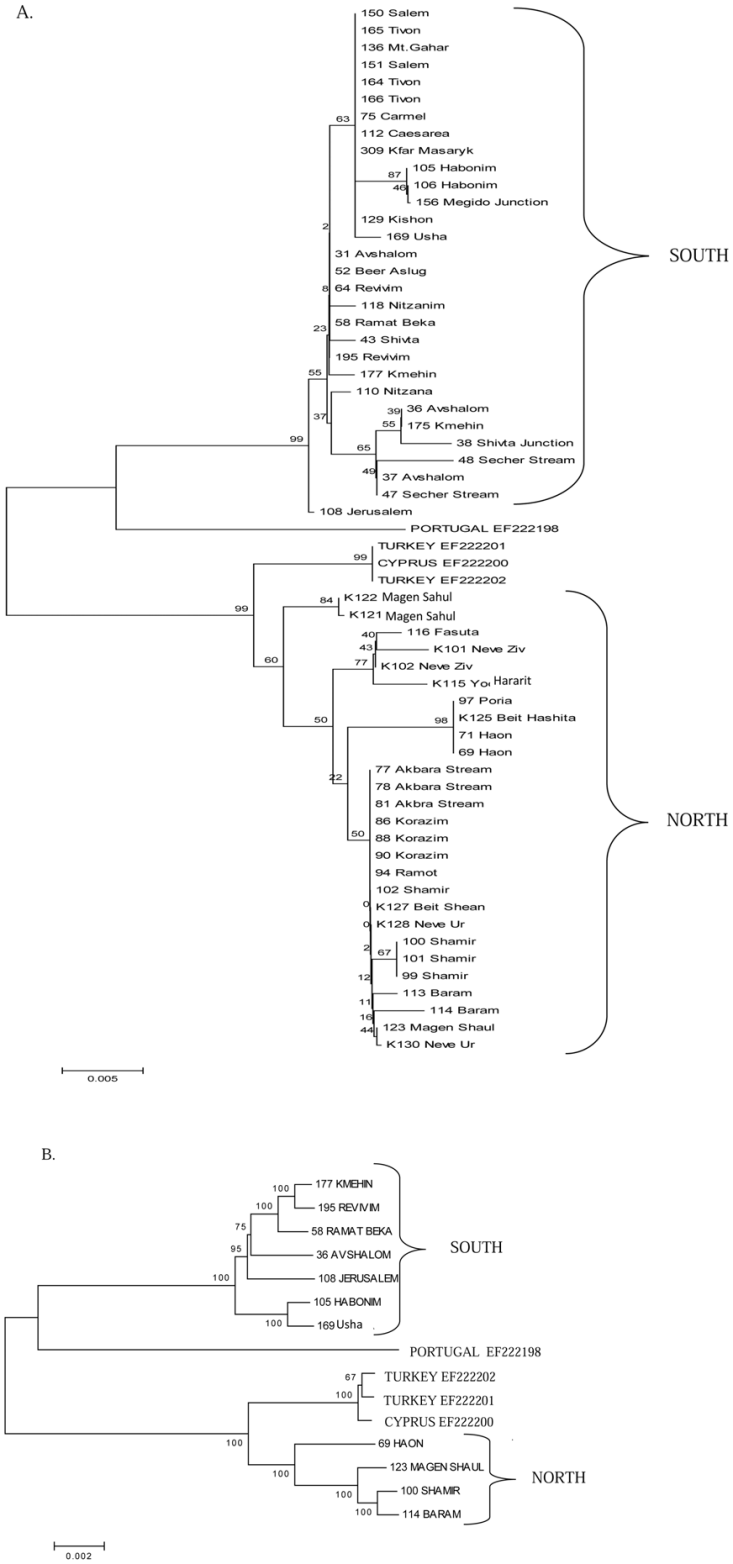
Neighbor joining trees based on mtDNA sequences from *C. chameleon* samples. (A) A tree constructed from 637 bp mtDNA fragments from 57 *C. chamaeleon* samples in Israel, one each from Portugal and Cyprus, and two from Turkey (Genbank accession numbers are shown). Collection sites are shown for each sample. To assess statistical significance the tree underwent a 1000 bootstrap replicates and scores (percentages) are mentioned near each branch. South –mtDNA genetic cluster including all samples south of the Jezreel Valley (see map in Fig 5). North –mtDNA genetic cluster including all samples north of the Jezreel Valley. For similar results using Network analysis – see Fig. S2. (B) A tree constructed from 11 whole mtDNA *C. chamaeleon* samples in Israel which were a subset of the samples analyzed in (A). Bootstrap scores of 1000 replicates are shown near each branch. All phylogenetic analyses were performed using MEGA 4.0.

**Figure 5:**
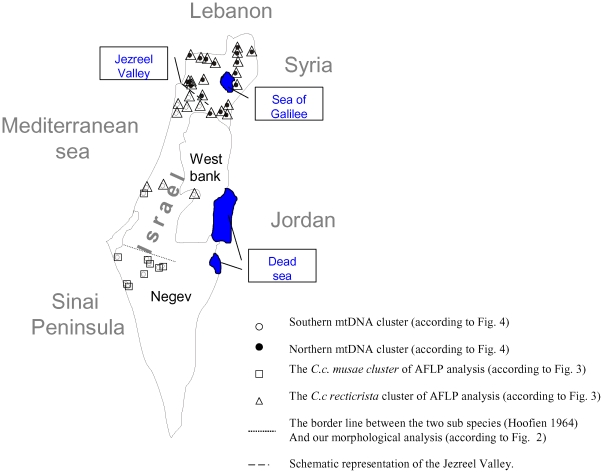
An integrative map summarizing the result of mDNA, AFLP and morphological analyses conducted for the 94 different chameleons sampled throughout Israel.

Sequence alignment and phylogenetic analysis of a 637 bp mtDNA fragment encompassing ∼2/3 of the ND2 gene, tRNATrp, and a non-coding sequence from a subset of 57 chameleon samples revealed two notably separated clusters that were strikingly different from the AFLP and morphological clustering (Fig. 4A, Fig. S2). One of the clusters included both the CCR and CCM subspecies sampled south of the Jezreel Valley encompassing the region from this valley to (and including) the Negev desert (Fig. 5), whereas the second cluster sampled north of the Jezreel Valley included only the CCR subspecies (Fig. 4A). Furthermore, the topology of this unconstrained phylogenetic mtDNA tree differed significantly from that of a constraint tree in which the separation between the two sub-species was imposed (Templeton test, N = 26, z = −4.5968, P<0.0001; Kishino-Hasegawa test, t = 4.9390, P<0.0001). This indicated that the division of mtDNA clusters across the Jezreel Valley was not related to the morphological differences between subspecies. AMOVA indicated that 79% of the genetic variation occurred between regions (northern and southern to the Jezreel Valley) while only 21% of the variation occurred among individuals within regions, implying significant genetic differentiation between regions (PhiPT = 0.79, p<0.001) (see Table 2). Also, PhiPT values between these two regions did not notably change when we performed the AMOVA separately for males (PhiPT = 0.86 p<0.001), and females (PhiPT = 0.83, p<0.001). This differentiation was well correlated with the ‘northern’ and ‘southern’ mtDNA clusters (Fig. 4). Coalescent-based estimation of effective migration rates (i.e., number of effective migrants per generation) indicated significant gene flow arrest between regions located north and south of the Jezreel Valley (θ_south_*Μ_south_ = 2.83e-12, θ_north_*Μ_north_ = 0.15) in both directions, although the strength of this effect appeared higher in the southbound direction. This result was in sharp contrast the AFLP results. Moreover, mtDNA variation was not correlated with the morphology-based taxonomic division of these subspecies. Closer inspection of both the phylogenetic mtDNA tree (Fig. 4A) and haplotype network (Fig. S2) illustrated a clear geographical subgrouping, especially in the southern mtDNA cluster. In addition, within the southern mtDNA cluster, specimens mapped to the geographical areas adjacent to Mount Carmel (mtDNA haplotypes H10, H12 and H14) formed a distinct sub-cluster compared to the rest of the southern specimens (Fig. S2). These haplotypes include only samples that were morphologically defined as belonging to the CCR subspecies, although mtDNA cluster analysis did not differentiate CCR from CCM overall. Mantel tests did not support correlation between genetic distance and geographical distance between samples collected north and south of the Jezreel Valley (Fig. S3A) even when we excluded CCM samples (Fig. S3B). Moreover, patterns of isolation by distance within each of these regions were not supported (Fig. S3C, D). Finally, while applying the Mantel test separately to the CCM samples (Fig. S3E), no isolation by distance was observed. Taken together isolation by distance could not explain any of the genetic variation patterns in *C. chamaeleon* in Israel.

**Table 2:** Inter and intra population mtDNA variation analyzed using AMOVA and PhiPT.*.

Whole mtDNA					
Source	Df	SS	MS	Est. Var.	%
Among Populations	1	1051.584	1051.584	197.326	81%
Within Populations	9	423.143	47.016	47.016	19%
Total	10	1474.727		244.342	100%

* AMOVA was performed and PhiPT was calculated as described in table 1. Column titles – as in Table 1. Among populations – between the regions ‘northern’ and ‘southern’ to the Jezreel Valley. Within populations – among individuals within regions.

In order to assess the extent of sequence divergence between the two major mtDNA clusters, we subjected 11 samples representing the two mtDNA clusters (6 ‘northern’ and 5 ‘southern’) to whole mtDNA sequence analysis. This analysis revealed 704 mutations dispersed throughout the mtDNA of which 325 (PhiPT = 0.808, p = 0.004) clearly separated the two mtDNA clusters (Fig. 4B). The remaining mutations (N = 379) comprised intra-population variation, mainly within the ‘southern’ mtDNA cluster (237 mutations). This magnitude of mtDNA sequence divergence drew our attention to the possibility that the two mtDNA clusters may be quite ancient (see below) and cryptic. Therefore, we compared whole mtDNA sequences from couples of the three closest ‘sister’ taxa of the genus *Chamaeleo* [16]. This revealed a mean of 990 mutations differentiating ‘sister’ taxa. Specifically, we found that the lowest number of mutations differentiating ‘sister’ *Chamaeleo* species was 551 SNP's (i.e., between the sister taxa *C. arabicus* and *C. calyptratus*); the differences among other sister taxa reached more than 1100 SNPs (i.e., between *C. chamaeleon* and *C. africanus*, and between *C. africanus* and *C. calyptratus*). Hence, we suggest that the number of mutations differentiating the ‘northern’ and ‘southern’ mtDNA clusters of chameleons in Israel is slightly less than the lowest degree of *Chamaeleo* species differentiation. This is consistent with an ancient divergence time, but is not likely to support an ancient taxonomic radiation.

We estimated the time of divergence between the two mtDNA clusters using Bayesian coalescence analysis in BEAST [18]. We analyzed the shorter 637 bp mtDNA fragment in the above mentioned 57 chameleons, using the publicly available mtDNA sequences of the Turkish and Cypriote *C. chamaeleon* as outgroups. This analysis suggested at least a 1 million year old divergence time (mean of 3.26 million years before present with a 95% HPD, i.e. Highest Posterior Density, with lower boundary at 0.95 and top boundary at 6.21 million years) of the two mtDNA clusters.

## Discussion

Our analysis of *C. chamaeleon* populations revealed a clear correlation between the geographic distribution of morphological characters and nuclear DNA data (AFLP). In contrast, a strikingly different pattern was evident in mtDNA sequence data. Previously observed analyses of the population genetic structure of vertebrate and invertebrate species has revealed inconsistencies between the geographic distribution of nuclear DNA and mtDNA markers. This has been attributed, in part, to different migration patterns of males and females [1]. AMOVA of the mtDNA genetic variability in *C. chamaeleon* populations supported divergence into groups collected north and south of the Jezreel Valley regardless of gender. This differentiation was well correlated with the mtDNA phylogenetic clustering. Therefore, *C. chamaeleon* mtDNA divergence across the Jezreel Valley was not likely due to differences between male and female migration patterns.

It is possible that genetic differentiation based on mtDNA and nuclear DNA-encoded markers are due to differences in mutation rates of the two genomes [12]. If mutational rate differences at least partially account for the lack of correlation between the mtDNA and AFLP cluster analyses of chameleons in Israel, traces of similarity would be expected between mtDNA clusters and the morphological divisions of chameleon subspecies. Within the ‘southern’ mtDNA cluster, specimens mapped to the geographical areas adjacent to Mount Carmel (mtDNA haplotypes H10, H12 and H14 in Fig. S2), all of which were defined morphologically as CCR, formed a distinct sub-cluster compared to the rest of the ‘southern’ specimens. However, mtDNA clustering did not differentiate CCR from CCM subspecies overall. Moreover, the number and nature of mtDNA mutations defining the ‘Mount Carmel area’ haplotypes was lower by an order of magnitude as compared to the number of mutations dividing the ‘southern’ and ‘northern’ mtDNA clusters. In addition, the Templeton test results underlined the fact that the mtDNA tree was significantly different from a tree that included the two named subspecies as monophyletic groups. Thus, we suspect that the Mount Carmel finding represents possible traces of differences in local adaptation of the CCR and CCM subspecies in southern Israel rather than a reflection of differences in mutation rates between nuclear and mitochondrial genomes. Clearly this interpretation requires further research.

How could one explain the lack of maternal gene flow, which is accompanied by more than 300 mtDNA SNPs differentiating the ‘northern’ from the ‘southern’ mtDNA clusters of *C. chameleon* in Israel? Could such a pattern be the sole result of random forces, namely differential genetic drift in male and females? The sharp divergence between the ‘southern’ and ‘northern’ mtDNA clusters does not reflect patches, as in previous studies, but rather long term divergence of the two groups supported by Bayesian coalescence (∼3 million years, lower limit of 1 million years). Being transmitted solely from the maternal side (in vertebrates), mtDNA variation is strongly influenced by a much smaller effective population size than nuclear DNA markers. However, mtDNA is very short (less than 20,000 bp in most vertebrates) and contains mostly coding sequences (∼90%) which are either essential protein components of the oxidative phosphorylation (OXPHOS) system or RNA encoding genes (2 rRNA and 22 tRNA) of the mitochondrial translational machinery. Thus it is not surprising that mtDNA sequences were reported to be subjected to negative or positive natural selection in various organisms [7], [19]–[21]. Natural selection could not only affect mitochondrial functions, but could alter their interactions with nuclear DNA factors that are imported into the mitochondria [22]. This latter cyto-nuclear interaction was shown to play a role in hybrid breakdown and adaptation to changing environments [23] and was suggested to be a major player in the formation of reproductive barriers and, eventually, in the emergence of new species [7]. PhiPT values, combined with a coalescent-based estimation of effective migration rates, supported the scenario of a nearly complete cessation of gene flow between the regions north and south of the Jezreel Valley, which correlated the two phylogenetic mtDNA clusters of chameleons in Israel. This finding is in sharp contrast to the general (AFLP) nuclear DNA markers. As mentioned above, such discrepancy has previously been attributed to the expected pattern of variation in hybrid zones [24]. However it is equally likely that mitochondrial-specific selective pressure shaped this pattern. It is worth noting that when we performed either the McDonald-Kreitman test or Ka/Ks assessment on the small number of whole mtDNA chameleon sequences, no clear traces for positive selection were detected in mtDNA genes (unpublished data). Nevertheless, we would like to stress that out of the 325 changes differentiating the ‘southern’ and ‘northern’ mtDNA clusters, 64 were amino acid replacements. This raises the possibility that at least a subset of these amino acid replacements affected the interaction of mtDNA-encoded subunits with nuclear DNA encoded subunits of OXPHOS complexes, as recently shown [25]. Clearly this possibility of cyto-nuclear interactions within the OXPHOS system should be further investigated.

As mentioned above, using a Bayesian coalescence analysis, we estimated the time of the most recent common ancestor of the two mtDNA genetic clusters and found values around ago. Our estimated divergence time of ca 3 millions of years coincides with the penetration of water from the Mediterranean Sea inland through the Jezreel Valley into the tectonic depression from the Sea of Galilee in the Pliocene to the present-day Dead Sea [26], [27]. Interestingly, the Jezreel Valley comprises the geographic border between the ‘northern’ and ‘southern’ mtDNA chameleon genetic clusters. It is possible that the divergence between the two mtDNA clusters was formed during the existence of this ancient geographic barrier and that the mtDNA mutation rate was fast enough to enable fixation of these variants.

In summary, our morphological results confirm the taxonomic assignment of two *C. chamaeleon* subspecies in the southern Levant and was consistent with data from nuclear DNA markers. In contrast, we found sharp divergence in mtDNA sequences resulting in two genetic clusters, the southern most of which harbors members of both morphological subspecies of *C. chamaeleon*. Since both mtDNA and nuclear DNA encoded markers supported gene flow between the two morphologically defined subspecies, there is indirect evidence for interbreeding. Lack of gene flow between the ‘northern’ and ‘southern’ mtDNA groups was is in sharp contrast to the patterns of nuclear DNA differentiation in this region, and suggests possible mitochondrial-specific selection. The existence of the ancient sea arm which created a border between the two mtDNA clusters suggests that separate accumulation of multiple mtDNA changes in the populations from either side of the border became fixed many years ago.

## Materials and Methods

### Sample collection

chameleons were located during night hours using a 4×4 vehicle equipped with strong spotlight projectors directed to shrubs and trees on which chameleons reside. Collection was made during several expeditions to various sites throughout Israel (see Fig. 1 for collection sites). Each of the captured chameleons was measured (Fig. S4), individually marked, photographed and released *in situ* after drawing blood (20–150 µl) from the caudal vein using 1 ml syringes. Blood was placed immediately in a 1.5 ml Eppendorf tube along with Gentra cell lysis solution according to manufacturer instructions. DNA was purified using the Puregene DNA extraction kit (Gentra, cat. # D-5500) following manufacturer instructions. The samples were collected in two datasets – the ‘genetic’ and ‘morphological’ datasets distributed as follows: 94 samples (70 females, 19 males, 5 juveniles) were analyzed for AFLPs (see below). Sequencing of a 637 bp mtDNA fragment was performed in a total of 57 individuals of which 35 were females, 19 males, and 3 juveniles. Of the samples subjected to mtDNA fragment sequencing 51 (35 females, 13 males, 3 juveniles) were also part of the AFLP analysis. In order to increase the knowledge about the mtDNAs clustering in the AFLP analysis, an additional 27 samples were analyzed using mtDNA RFLP to define the ‘southern’ and ‘northern’ mtDNA clusters (see below). A total of 147 *C. chamaeleon* specimens were collected throughout Israel and subjected to morphological analysis of which 31 were also included in the above mentioned genetic analysis (both AFLP and mtDNA). These specimens included 125 *C. c. recticristae* (50 males, 75 females) and 20 *C. c. musae* (7 males, 13 females) as well as two juveniles. All chameleon samples were collected using permits from the Israel authority of nature preservation, numbers 2007/30542, 2008/31815, 2008/31819, 2010/37685.

### Morphological analysis

size measurements of each chameleon were conducted photographically using a millimetric paper background. Fourteen characters were measured: (1) crest head height, (2) crest–mouth length, (3) jaw length, (4) dorsal crest length, (5) dorsal crest–mouth, (6) dorsal crest width, (7) elbow-hand joint, (8) armpit-elbow, (9) digitus tertius, (10) annulus finger, (11) foot digitus tertius, (12) foot annulus finger, (13) eye vertical diameter and (14) eye horizontal diameter. The chameleons were photographed (Fig. S4 in the supplementary material) using a tripod (where S1 = 120 cm), Tele Macro Lens in bright day light for Maximum Aperture value (closed aperture) in order to increase the depth of field.

The chameleon pictures were measured digitally using the millimeter paper background as size calibration. In order to correctly measure our objects, we took into account perspective distortion (Fig. S4), i.e. erroneous size differences of objects with identical sizes that are positioned at different distances from the camera (Fig. S4). In order to determine the perspective distortion effect (Fig. S4) we used the following equation:
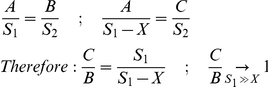
where S1 and S2 correspond to the distance of object 1 and the light sensitive camera cell to the focal center, respectively. Object 1 corresponds to the millimeter paper and Object 2 represents the chameleon being measured. All trait values were log-transformed prior to statistical analyses. We used multivariate analysis of variance (MANOVA) followed by canonical discriminant function analysis [28] to test for morphological differences in size and shape between the two subspecies. MANOVA enabled us to test for significant differences between subspecies while considering all traits simultaneously. The second analysis enabled us to find linear combination of morphological traits best separating the subspecies defined by all the morphological traits considered. In addition, we used Principal Component Analysis (PCA) [28] to generate two composite variables from all 14 morphological traits measured, describing overall chameleon body size (PC1) in comparison to the proportions of the different traits (PC2), i.e., shape differences independent of body size.

### MtDNA amplification and sequencing

we amplified and sequenced a 637 bp fragment from 57 chameleons (including the 3′ end of ND2, the 5′ end of tRNA-Trp, positions 4278–4914 according to Genbank accession number EF222202.1). Using the annotation specified in EF222202.1 we amplified and sequenced the whole mtDNA of 11 chameleons (∼17400 bp in each) in five overlapping fragments using a set of five primer pairs (Table S3 and Fig. S5), each in a 50 µl reaction volume (apart from fragments 2 and 3b). Final reaction concentrations were: 0.1 U/µL Taq polymerase (Finnzymes) , Taq buffer ×1, 2 mM MgCl_2_, 2 mM dNTP's, 200 nM of each of forward and reverse primers (Table S8), 100 ng DNA template and sterile double distilled water added up to 50 µL of the total reaction volume.

Fragment 1a (3300 bp) was amplified using the following program: Denaturation for 5 minutes at 94°C followed by 35 cycles including 30 seconds at 94°C, 1 minute annealing at 58°C, 4 minutes elongation at 72°C. After a final 7 minutes elongation step at 72°C, the reaction was concluded at 15°C and kept frozen at −20°C until use.

Fragment 1b (3700 bp) was amplified using the following program: Denaturation for 5 minutes at 94°C followed by 40 cycles including 30 seconds at 94°C, 1 minute annealing at 64°C, 4 minutes elongation at 72°C. After completing the 40 cycles, a final 7 minutes elongation step at 72°C was performed and the reaction was concluded at 15°C and kept frozen at −20°C until use.

Fragment 2 (5700 bp) was amplified in a 40 µl reaction volume with final concentration were 0.016 U/µL Phusion polymerase, ×1 HF buffer (provided with the enzyme), 1.6 mM dNTP's, 1 µL of 10 µM forward primer (200 nM), 0.25 µM reverse primer, 100 ng DNA template and sterile water added up to 40 µL of the total reaction volume. The amplification was performed using the following polymerase chain reaction (PCR) program: Denaturation for 5 minutes at 94°C followed by 35 cycles including 30 seconds at 94°, 1 minute annealing at 55°C, 6 minutes 30 seconds elongation at 72°C. After a final 7 minutes elongation step at 72°C the reaction was concluded at 15°C and kept frozen at −20°C until use.

Fragment 3a (3700 bp) was amplified using the following PCR program: Denaturation for 5 minutes at 94°C followed by 35 cycles including 30 seconds at 94°C, 1 minute annealing at 60°C, 4 minutes elongation at 72°C. After a final 7 minutes elongation step at 72°C the reaction was concluded at 15°C and kept frozen at −20°C until use.

Fragment 3b (3500 bp) was amplified in a 40 µl reaction volume using 0.4 µL Phusion polymerase, 8 µL HF buffer ×5, 3.2 µL 25 mM dNTP's, 1 µL of 10 µM primer F, 1 µL of 10 µM primer R, 100 ng DNA template and water up to 40 µL total volume. The PCR program was as follows: Denaturation for 5 minutes at 94°C followed by 35 cycles including 30 seconds at 94°C, 1 minute annealing at 68.3°C, 4 minutes elongation at 72°C. After the final 7 minutes elongation step at 72°C the reaction was concluded at 15°C and kept frozen at −20°C until use.

Following purification (Promega wizard SV Gel and PCR clean-up system), the amplified whole mtDNA fragments of 11 chameleons were sequenced using a set of 49 primers (Table S4). The sequencing reactions were carried out using the following PCR conditions: Denaturation for 2 minutes at 95°C followed by 35 cycles including 30 seconds at 96°C, 30 seconds annealing at 50°C, 4 minutes elongation at 60°C. Reaction was concluded at 10°C and was then sequenced in a 3130xl Genetic Analyzer (Applied Biosystem- HITACHI).

### DNA sequence and phylogenetic analyses

multiple sequence alignment for initial viewing was performed using Sequencher 4.1 (Gene Codes Corporation, MI USA). Multiple sequence alignment and phylogenetic analysis was performed using MEGA 4.0 [29]. Similar tree topologies were obtained using three different types of phylogenetic methods: (A) Neighbor joining, Bootstrap (1000 replicates), Gap/missing data – pairwise deletion, Model – Maximum composite likelihood, Substitution to include – d: Transitions+transversions, Pattern among Lineages – homogeneous, Rate among sites – Uniform rates; (B) Maximum Likelihood, Bootstrap (1000 replicates), Gap/missing data – Use all sites, Model – Tamura-Nei model, Substitution type – Nucleotide, Rate among sites – Uniform rates, ML Heuristic method – NNI, automatic initial tree for ML; (C) Maximum Likelihood with HKY model with Gamma distribution among sites (also used for BEAST analysis). To test if the topology of this unconstrained phylogenetic tree differed significantly from that of a constraint tree in which a separation between the two sub-species was imposed, we used the Kishino-Hasegawa [30] and Templeton tests [31]. Specifically, we first used MacClade v 4.03 to create a constraint tree in which the separation between the two sub-species was imposed. We imported the tree to PAUP v. 4b10 and performed a thorough ML tree search with the constraint imposed. Doing so generated the likeliest tree which meets this constraint. We then loaded both the constraint and unconstraint trees and contrasted their topologies using the Kishino-Hasegawa [30] and Templeton tests [31] as implanted in PAUP v. 4b10.

### Testing for isolation by distance

we performed a Mantel test using GenAlex, ver. 6.3 [32] allowing for 9999 permutations. The test compared the matrix of pair-wise genetic distances with that of geographical distances.

### Testing for genetic differentiation in the mtDNA data

analysis of molecular variance (AMOVA) was performed using GenAlex, ver. 6.3 [32]. In the frame of the mtDNA analysis the samples were divided according to those collected north and south of the Jezreel Valley. The sequences were transformed into Nei's genetic distance table. This table was then used for AMOVA with the following parameters: number of Samples - 57, number of loci – 50 (variables sites), number of populations – 2, number of permutations 9999.

We also performed AMOVA using the 11 whole mtDNA sequences dividing the samples according to those samples north and south of the Jezreel Valley. The sequence was transformed into Nei's genetic distance table. This table was then used for AMOVA with the following parameters: number of Samples - 11, number of loci – 748 (variables sites), number of populations – 2, number of permutations 9999.

### Estimating effective migration rates

using Migrate 3.2.15 we obtained a coalescent-based estimation of effective migration rates (i.e., number of effective migrants per generation). This computer program uses a Markov chain Monte Carlo (MCMC) approach with importance sampling [33] to estimate θ×Μ, where θ is the product of effective population size (N_e_), mutation rate per site per generation (μ) and inheritance parameter (x; x = 1 for mtDNA data), and M is the proportion of individuals migrating per generation (*m*) divided by μ. We used a Maximum Likelihood search strategy with 20 short chains, 10,000 recorded steps, 1,000,000 visited genealogies, 2 long chains with 100,000 recorded steps, and 10,000,000 visited genealogies. In both chains the burn in period was 10,000 trees. We assumed a constant mutation rate, variable M and that θ is the same for all populations. Similar results were obtained when using different mutation rates (relative) in combination with variable θ and symmetric M as previously described [33].

### Amplified Fragment Length Polymorphism (AFLP)

We performed AFLP on 94 chameleons, of which 78 were also subjected to mitochondrial DNA analysis (51 out of 57 sequences from Fig. 4a having 637 bp mtDNA sequence and 27 with RFLP – see Table S5). The AFLP procedure was performed as previously described by Shaked et al. [34], with minor modifications. In brief, 0.5 µg of the extracted genomic DNA of each of the samples was digested at 37°C for 2 hr using EcoR1 and MseI (NEB). Each reaction mix contained both restriction enzymes and a DNA ligase as follows: 0.12 µl of 10000 u/ml MseI (New England Biolabs, NEB), 0.5 µl of 20000 u/ml EcoRI (NEB), 0.3 µl of 400000 u/ml T4-DNA ligase (NEB), 0.5 M NaCl, 1 µl of BSA (1 mg/ml), and 1 µl of 10× DNA ligase buffer (NEB), Mse1 adaptor pair oligos (16 µg of adaptor 1 and 14 µg of adaptor 2, diluted with sterile water to a total volume of 60 µl), EcoR1 adaptors pair oligos (1.7 µg adaptor 1, 1.5 µg of adaptor 2 diluted with double distilled water (DDW) to a final volume of 60 µl) in a final volume of 1 µl each. The digested-ligated DNA was diluted 1∶10 with 90 µL of sterile double-distilled water. The adaptor sequences were as follows: MseI adaptor 1#- 5′-TACTCAGGACTCAT-3′ and adaptor 2# 5′-GACGATGAGTCCTGAG-3′; EcoRI adaptor 1#- 5′-CTCGTAGACTGCGTACC-3′ and adaptor 2#- 5′-AATTGGTACGCAGTCTAC- 3′.

Pre-selective amplification was performed using primers complementary to the core of the adaptor sequences; the EcoRI pre-selective primer was 5′-GACTGCGTACCAATTCA-3′, and the MseI pre-selective primer was 5′-GATGAGTCCTGAGTAAC-3′. The PCR reaction mixture contained the following: one tenth of the reaction volume corresponds to the restricted-ligated DNA, 50 ng EcoRI pre-selective primer, 50 ng MseI pre-selective primer, 1.5 unit of Taq DNA polymerase (Biolabs), 2 µL of 10× Taq DNA polymerase buffer (Biolabs) supplemented by 2 µL of 25 mM MgCl_2_, and 0.8 µL of 2.5 mM dNTPs mix in a final volume of 20 µL. The PCR program was: 94°C for 30 sec followed by 30 cycles of 30 sec at 94°C, 30 sec at 56°C, and 1 min at 72°C. After pre-amplification, the PCR products were diluted 1∶20 with 190 µL of double-distilled water.

Selective amplification was performed using a FAM labeled EcoRI selective primer 5′-GACTGCGTACCAATTCACT-3′ and the unlabeled MseI selective primer 5′-GATGAGTCCTGAGTAACTG-3′. The selective amplification reaction contained 50 ng of template DNA from the pre-selective amplification, 1 ng labeled EcoRI selective primer, 5 ng MseI selective primer, 1 unit Taq DNA polymerase (Bioloabs), 2 µL of Taq DNA polymerase buffer (Biolabs), 2 µL of 25 mM MgCl_2_, and 2.5 mM dNTP mix in a final volume of 20 µL. The reactions were placed in a MJ (BioRad) PCR machine and were subjected to one cycle of 2 min at 94°C, 30 sec at 65°C, and 1 min at 72°C, followed by 10 cycles of 30 sec at 94°, 30 sec at 64°C, 72° for 1 min. These cycles were followed by 27 cycles of 30 sec at 94°C, 30 sec at 56°C, and 1 min at 72°C. When completed, the reaction was kept on ice and 2.5 µL of the selective product were loaded onto 96 wells plate (Applied Biosystems) with each well containing 9.9 µL 98% formamide and 0.6 ROX size standards (Applied Biosystems). The samples were then loaded on 3130xl Genetic Analyzer (Applied Biosystem- HITACHI).

The results were analyzed using GeneMapper V4.0 (Applied Biosystems) that generated an alleles table. This table was then used for creating Nei's genetic distance table. This table was then used for analysis of molecular variance (AMOVA) with the following parameters: number of Samples - 94, number of loci – 323, number of populations – 2, number of permutations 999. We performed AMOVA and Principle Coordinate Analysis (PCA) using GenAlex, ver. 6.3 [32].

Three AFLP profiles were re-run starting from the digestion phase in order to ensure reproducibility of the resulting profiles.

### PCR-RFLP

Sequence and phylogenetic analysis of a ∼637 bp mtDNA fragment in 57 chameleons revealed two clearly separate clusters. This clear separation enabled us to identify restriction sites indicative of the two clusters. These sites were used to screen for additional chameleon samples and assign them to either of the two mtDNA clusters. We identified two such restriction sites. First, we PCR amplified an 894 bp mtDNA fragment encompassing the identified restriction sites using the following primers: F-5′GCTGCCCCAATTTACTTCTG′ and R-5′ATCAAGGCCTACTAGTCCTG3′ (nucleotide positions 4148-4167 and 5042-5023, Genbank accession number EF222202.1, [16]). The fragment was digested using AseI (BioLabs cat # R0526S) and BsmAI (BioLabs cat # R0529S) in two separate reactions (Table S5).

### Estimation of most recent common ancestor

We used a Bayesian coalescence framework to simulate the time of divergence of the last common ancestor of both ‘southern’ and ‘northern’ mtDNA clusters. We used the partial mtDNA sequences (637 bp) for two reasons: (A) Since PhiPT values were based on this fragment and the entire mtDNA genome were very close, their mutation rates were probably similar. (B) In order to estimate divergence times with confidence, we aimed at the largest sample size available. Hence, we preferred using the 637 bp mtDNA fragment for which 57 samples were available as opposed to 11 samples encompassing the whole mtDNA. Specifically, we estimated the time of divergence using the ‘birth and death’ speciation model, implemented in BEAST [18]. This method takes into account both the error inherent in phylogenetic reconstruction and the stochastic error intrinsic to the speciation process, and thus produces more correct estimates of statistical uncertainty. In addition, the MCMC sampling procedure implemented in BEAST allows for simultaneous estimation of the ancestral genealogy, and parameters of the substitution process and demography. Sequences were simulated down the tree using the Hasegawa, Kishino and Yano (HKY) model and empirical base frequencies, while also allowing for a heterogeneous (four-category gamma distribution) mutation rate among sites (HKY and gamma distribution models were chosen using a model selection procedure in J model 0.1.1) [35]. MCMC chains were run for 10,000,000 iterations of which the first 10% were discarded to allow for burn-in. Genealogies and model parameters were sampled per 1,000 iterations. We assumed an uncorrelated lognormal relaxed molecular clock model, and estimated the divergence time of the two mtDNA chameleon entities using previously reported divergence rate data of chameleons [36].

## Supporting Information

Figure S1 **The distribution of genders appears uniform within each taxon.** Upper panel: The dashed line separates the CCR (down right) from the CCM (upper left) taxa. Circles- females; X – males; + - juvenile specimen. The two sub species (CCR and CCM) are separated mainly along the second principal axis. PC1 describes the average ontogenetic allometry of individuals from both subspecies. The CCM adults are located left on the size axis (PC1) indicating that they are smaller than the CCR adults. The separation along the PC2 axis is due to differences in the shape of the chameleons which are independent of their body size. Notice that the distribution of males and females is indistinguishable among the two taxa. The single point within the lower left corner is the only juvenile in the analysis. This point emphasizes the separation between the taxa mainly by shape and not in size. Lower panel: The canonical discriminant function analysis of the morphological data set, using subspecies as a grouping variable. Dashed line separates CCR (left of line) from CCM (right of line). Again, the observed distribution of genders is uniform within each taxon and it is clear that the taxa separation is not due to sexual dimorphism in size or shape. MANOVA indicated that there are significant difference in morphology between subspecies (Wilk's Lambda = 0.161, P = 0.000, df = 84, 697). A canonical discriminant function analysis on morphology provided an expression for the maximal separation of the original two sub species. Based on this expression, the trait best suited to discriminate between them is the foothold finger size (parameter K, Table S1) (F-to-remove = 3.27), and the second most indicative trait is the arm size (parameter I, Table S1) (F-to-remove = 2.32). The canonical variate (standardized by within-group variances), best separating between the sub-species is: CV1 = 0.345×logA×0.834×logB−0.013×logD−0.03×logE+0.257×logF+0.121×logG−0.91×logH+0.371×logI−0.45×logJ−1.49×logK+0.354×logL−1.043×logM−0.609×logN+0.83×logO.
(DOC)

Figure S2 **Haplotype network analysis according to sequence alignment of the 637 bp mtDNA fragment obtained from** ***C. chamaeleon*** **samples.** The results were obtained using Network 4.516, by aligning the 637 bp coding region mtDNA fragment (see main text). The network analysis indicates the changes between each haplotype and its frequency in the analyzed population of Israeli chameleons as well as in two available Turkish, one Cypriote and one Portuguese chameleon sequences of the same species (*Chamaeleo chamaeleon*). The collection sites of each of the Israeli chameleons are mentioned. Southern mtDNA cluster – samples mapped southern to the Jezreel Valley. Northern mtDNA cluster - samples mapped northern to the Jezreel Valley. Hap_1: 1 [PORTUGAL_EF222198], Hap_2: 3 [TURKEY_EF222202 TURKEY_EF222201 CYPRUS_EF222200], Hap_3: 6 [31_Avshalom 52_Beer_Aslug 58_Ramat_Beka 64_Revivim 195_Revivim 43_Shivta], Hap_4: 2 [36_Avshalom 175_Kmehin], Hap_5: 2 [37_Avshalom 47_Secher], Hap_6: 1 [38_Shivta], Hap_7: 1 [48_Secher], Hap_8: 1 [110_Nitzana], Hap_9: 1 [177_Kmehin], Hap_10: 10 [75_Carmel 112_Caesarea 129_Kishon 136_Mt.Gahar 150_Salem 151_Salem 164_Tivon 165_Tivon 166_Tivon 309_Kfar_Masaryk], Hap_11: 1 [108_Jerusalem], Hap_12: 3 [105_Habonim 106_Habonim 156_Megido_Junction], Hap_13: 1 [118_Nitzanim], Hap_14: 1 [169_Oosha], Hap_15: 4 [69_Haon 71_Haon 97_Poria K125_Beit_Hashita], Hap_16: 12 [77_Akbara_Stream 78_Akbara_Stream 81_Akbra_Stream 86_Korazim 88_Korazim 90_Korazim 94_Ramot 102_Shamir 123_Magen_Shaul K127_Beit_Shean K130_Neve_Ur K128], Hap_17: 3 [99_Shamir 100_Shamir 101_Shamir] ,Hap_18: 1 [113_Baram], Hap_19: 1 [114_Baram], Hap_20: 1 [116_Fasuta], Hap_21: 1 [K101_Neve_Ziv], Hap_22: 1 [K102_Neve_Ziv], Hap_23: 1 [K115_Yodfat], Hap_24: 2 [K122_Afula K121_Afula].
(DOC)

Figure S3 **Mantel test: Genetic Distance (GD) vs Geographic Distance (GGD).** Four analyses which test for possible isolation by distance were performed: A. Between the samples collected north and south of the Jezreel Valley - Rxy = 0.359, *P* = 0.000, R^2^ = 0.1292, based on 9999 permutations. B. Between the samples collected northern and southern to the Jezreel Valley excluding the CCM subspecies, Rxy = 0.412, *P* = 0.000, R^2^ = 0.1699, based on 999 permutations. C. In the samples collected southern to the Jezreel valley (in order to detect intra-cluster isolation by distance) Rxy = 0.360, *P* = 0.000, R^2^ = 0.1295, based on 9999 permutations. D. In the samples collected northern to the Jezreel Valley (same reason as C) Rxy = 0.397, *P* = 0.000, R^2^ = 0.1576, based on 9999 permutations. E. only in the CCM sub species, Rxy = 0.046, P = 0.298, R^2^ = 0.0021, based on 9999 permutations.
(DOC)

Figure S4
**Morphological measurements of the chameleons.** Morphology size measurements conducted by chameleon Photography over a millimetric paper background. The chameleons were photographed (A) using a tripod (where S1 = 120 cm), Tele Macro Lens, and in bright day light for maximum aperture value (closed aperture) in order to increase the depth of field. (B) Skull measurements. (C–D) Measurements of other body parts. (E) Perspective distortion effect. The chameleon portrait was digitally measured using the background as size calibration. Perspective distortion generated by the distance *X* cause *Figure 2* look bigger then *Figure 1* although both represent objects with identical sizes. From the geometric principle of similarity of triangles one can state that :
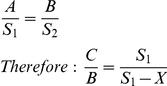
where *Object 1* represents the millimetric paper background and *Object 2* is the chameleon being measured. Perspective distortion effect was tested using these characteristics (with different X sizes), as shown in (F). Example: for a 3 cm wide chameleon (*X = 3 cm*) we would expect less then 3% size distortion, which was defined as sufficient accuracy. For detailed explanation – see Materials and Methods.(DOC)

Figure S5 **Schematic map of the mitochondrial genome of** ***C. chamaeleon*** **.** Arrows designate primers used for PCR amplification of whole mtDNA in 5 overlapping fragments (for primers sequences and nucleotide positions – see Table 1. Genes were assigned according to the annotation within whole mtDNA sequence of a Turkish *C. Chamaeleon* (Genbank accession number EF222202.1). Genbank accession numbers were assigned to 57 *C. chameleon* mtDNA sequences: Forty six 637 bp mtDNA fragment sequences and 11 whole mtDNA sequences. Following are the accession numbers of the 46 short (637 bp) mtDNA fragments: JF317646 (31_Avshalom), JF317647 (37_Avshalom), JF317648 (38_Shivta_Junction), JF317649 (47_Secher_Stream), JF317650 (48_Secher_Stream), JF317651 (52_Beer_Aslug), JF317652 (64_Revivim), JF317653 (110_Nitzana), JF317654, (75_Carmel), JF317655 (106_Habonim), JF317656 (112_Caesarea), JF317657 (118_Nizzanim), JF317658 (129_Kishon), JF317659 (136_Mt.Gahar), JF317660 (150_Salem), JF317661 (151_Salem), JF317662 (164_Tivon), JF317663 (165_Tivon), JF317664 (71_Haon), JF317665 (77_Akbara_Stream), JF317666 (78_Akbara_Stream), JF317667 (81_Akbra_Stream), JF317668 (86_Korazim), JF317669 (88_Korazim), JF317670 (90_Korazim), JF317671 (94_Ramot), JF317672 (97_Poria), JF317673 (99_Shamir), JF317674 (101_Shamir), JF317675 (102_Shamir), JF317676 (113_Baram), JF317677 (116_Fasuta), ), JN830601 (Kmehin), JN830602 (Tivon), JN830603 (Megido_Junction), JN830604 (Shivta), JN830605 (Kfar Masaryk), JN830606 (Neve_Ziv), JN830607 (Neve_Ziv), JN830608 (Beit Shean), JN830609 (Hararit), JN830610 (Magen_Shaul), JN830611 (Magen_Shaul), JN830612 (Beit_Hashita), JN830613 (Neve Ur), JN830614 (Neve Ur). Genbank accession numbers were assigned to 11 whole mtDNAs of chameleon in Israel: JF317635 (36_AVSHALOM), JF317636 (58_RAMAT_BEKA), JF317637 (177_KMEHIN), JF317638 (195_REVIVIM), JF317639 (105_HABONIM), JF317640 (108_JERUSALEM), JF317641 (169_OOSHA), JF317642 (100_SHAMIR), JF317643 (69_HAON_EAST_OF_KINERET), JF317644 (114_BARAM), JF317645 (123_MAGEN_SHAUL).
(DOC)

Table S1 **The normalized principal component loadings with coefficient intervals calculated from 1000 bootstrap.** Variables A,B,D,E,F,G,N,O are head traits while H,I,J,K,L,M are limbs traits.
(DOC)

Table S2 **Stepwise discriminant function analysis.** Six parameters of head and limbs traits define the minimal function needed to distinguish between the two subspecies. Wilks' lambda = 0.214, df = 36,582 P<0.001. The minimal canonical variant (standardized by within-group variances), required for best separating between the sub-species is: CV1 = 1.018×logB+0.256×logE+0.404×logI−1.386×logK−0.902×logM+0.379×logO.
(DOC)

Table S3 **Primers list (F-Forward, R-Reverse) for whole mtDNA amplification.** Nucleotide positions were assigned using the whole mtDNA sequence of a Turkish Chamaeleo Chamaeleon (Genbank accession number EF222202.1). For the content of mt genes in each fragment see Figure S5.
(DOC)

Table S4 **List of primers used to sequence the whole chameleon mtDNA.** Nucleotide positions were assigned using the whole mtDNA sequence of a Turkish Chamaeleo Chameleon (Genbank accession number EF222202.1).
(DOC)

Table S5 **mtDNA RFLP screen of** ***C. chamaeleons*** **in Israel.**
(DOC)
