# Supplementary material for: Mitochondrial DNA Variation, but Not Nuclear DNA, Sharply Divides Morphologically Identical Chameleons along an Ancient Geographic Barrier
Source: PLoS One. 2012 Mar 13;7(3):e31372. doi: 10.1371/journal.pone.0031372 (PMC3306244; doi:10.1371/journal.pone.0031372)
Supplement: Figure S1 — The distribution of genders appears uniform within each taxon. Upper panel: The dashed line separates the CCR (down right) from the CCM (upper left) taxa. Circles- females; X – males; + - juvenile specimen. The two sub species (CCR and CCM) are separated mainly along the second principal axis. PC1 describes the average ontogenetic allometry of individuals from both subspecies. The CCM adults are located left on the size axis (PC1) indicating that they are smaller than the CCR adults. The separation along the PC2 axis is due to differences in the shape of the chameleons which are independent of their body size. Notice that the distribution of males and females is indistinguishable among the two taxa. The single point within the lower left corner is the only juvenile in the analysis. This point emphasizes the separation between the taxa mainly by shape and not in size. Lower panel: The canonical discriminant function analysis of the morphological data set, using subspecies as a grouping variable. Dashed line separates CCR (left of line) from CCM (right of line). Again, the observed distribution of genders is uniform within each taxon and it is clear that the taxa separation is not due to sexual dimorphism in size or shape. MANOVA indicated that there are significant difference in morphology between subspecies (Wilk's Lambda = 0.161, P = 0.000, df = 84, 697). A canonical discriminant function analysis on morphology provided an expression for the maximal separation of the original two sub species. Based on this expression, the trait best suited to discriminate between them is the foothold finger size (parameter K, Table S1) (F-to-remove = 3.27), and the second most indicative trait is the arm size (parameter I, Table S1) (F-to-remove = 2.32). The canonical variate (standardized by within-group variances), best separating between the sub-species is: CV1 = 0.345×logA×0.834×logB−0.013×logD−0.03×logE+0.257×logF+0.121×logG−0.91×logH+0.371×logI−0.45×logJ−1.49×logK+0.354×logL− [file pone.0031372.s001.doc]

-10

-5

0

5

PC1

-3

-2

-1

0

1

2

3

4

PC2

Juv

M

F

-4

-3

-2

-1

0

1

2

3

4

5

6

CV1

-4

-3

-2

-1

0

1

2

3

CV2

Juv

M

F
