# Supplementary material for: Mitochondrial DNA Variation, but Not Nuclear DNA, Sharply Divides Morphologically Identical Chameleons along an Ancient Geographic Barrier
Source: PLoS One. 2012 Mar 13;7(3):e31372. doi: 10.1371/journal.pone.0031372 (PMC3306244; doi:10.1371/journal.pone.0031372)

Portuguese chameleon

Southern mtDNA cluster

Turkish and Cypriote chameleons

Northern mtDNA cluster population


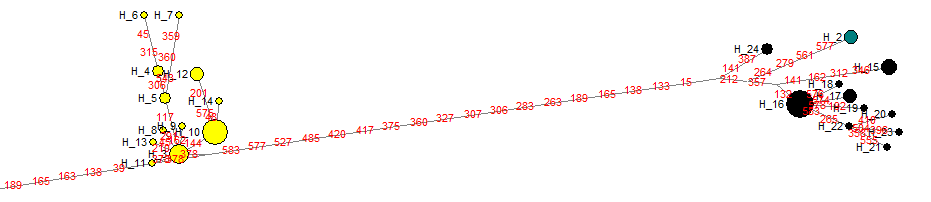


Southern mtDNA cluster

Northern mtDNA cluster


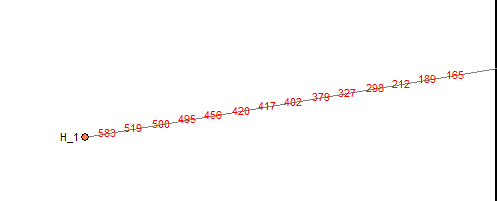

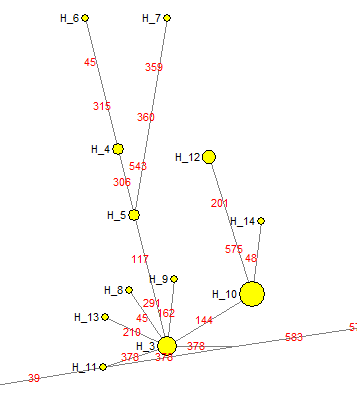

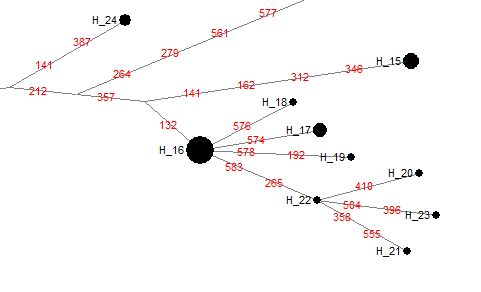

Supplement: Figure S2 — Haplotype network analysis according to sequence alignment of the 637 bp mtDNA fragment obtained from C. chamaeleon samples. The results were obtained using Network 4.516, by aligning the 637 bp coding region mtDNA fragment (see main text). The network analysis indicates the changes between each haplotype and its frequency in the analyzed population of Israeli chameleons as well as in two available Turkish, one Cypriote and one Portuguese chameleon sequences of the same species (Chamaeleo chamaeleon). The collection sites of each of the Israeli chameleons are mentioned. Southern mtDNA cluster – samples mapped southern to the Jezreel Valley. Northern mtDNA cluster - samples mapped northern to the Jezreel Valley. Hap_1: 1 [PORTUGAL_EF222198], Hap_2: 3 [TURKEY_EF222202 TURKEY_EF222201 CYPRUS_EF222200], Hap_3: 6 [31_Avshalom 52_Beer_Aslug 58_Ramat_Beka 64_Revivim 195_Revivim 43_Shivta], Hap_4: 2 [36_Avshalom 175_Kmehin], Hap_5: 2 [37_Avshalom 47_Secher], Hap_6: 1 [38_Shivta], Hap_7: 1 [48_Secher], Hap_8: 1 [110_Nitzana], Hap_9: 1 [177_Kmehin], Hap_10: 10 [75_Carmel 112_Caesarea 129_Kishon 136_Mt.Gahar 150_Salem 151_Salem 164_Tivon 165_Tivon 166_Tivon 309_Kfar_Masaryk], Hap_11: 1 [108_Jerusalem], Hap_12: 3 [105_Habonim 106_Habonim 156_Megido_Junction], Hap_13: 1 [118_Nitzanim], Hap_14: 1 [169_Oosha], Hap_15: 4 [69_Haon 71_Haon 97_Poria K125_Beit_Hashita], Hap_16: 12 [77_Akbara_Stream 78_Akbara_Stream 81_Akbra_Stream 86_Korazim 88_Korazim 90_Korazim 94_Ramot 102_Shamir 123_Magen_Shaul K127_Beit_Shean K130_Neve_Ur K128], Hap_17: 3 [99_Shamir 100_Shamir 101_Shamir] ,Hap_18: 1 [113_Baram], Hap_19: 1 [114_Baram], Hap_20: 1 [116_Fasuta], Hap_21: 1 [K101_Neve_Ziv], Hap_22: 1 [K102_Neve_Ziv], Hap_23: 1 [K115_Yodfat], Hap_24: 2 [K122_Afula K121_Afula]. (DOC) [file pone.0031372.s002.doc]
