# Supplementary figures and images for: Mitochondrial DNA Variation, but Not Nuclear DNA, Sharply Divides Morphologically Identical Chameleons along an Ancient Geographic Barrier
Source: PLoS One. 2012 Mar 13;7(3):e31372. doi: 10.1371/journal.pone.0031372 (PMC3306244; doi:10.1371/journal.pone.0031372)

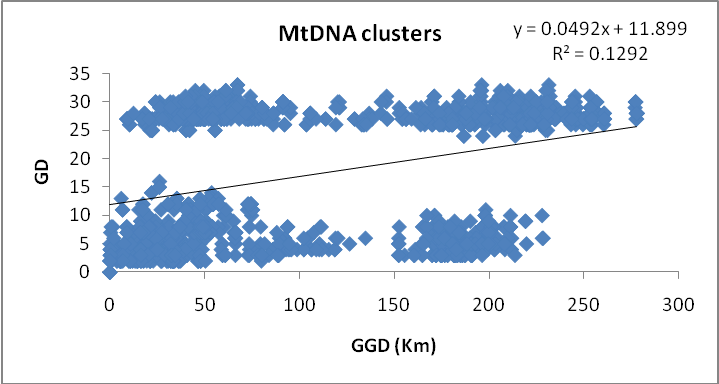

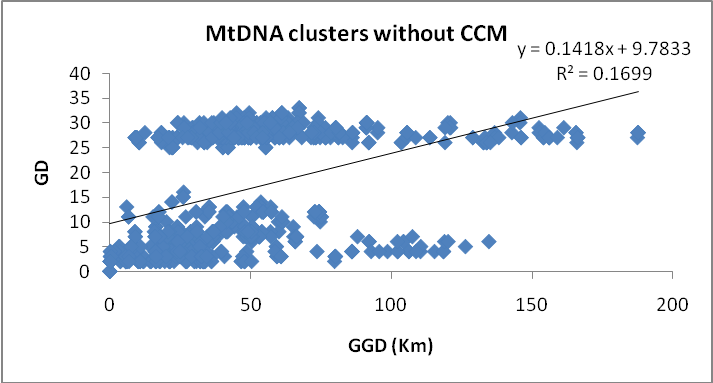


**D**

**B**

**C**

**A**


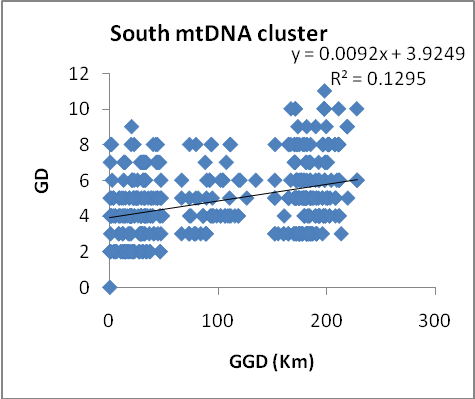

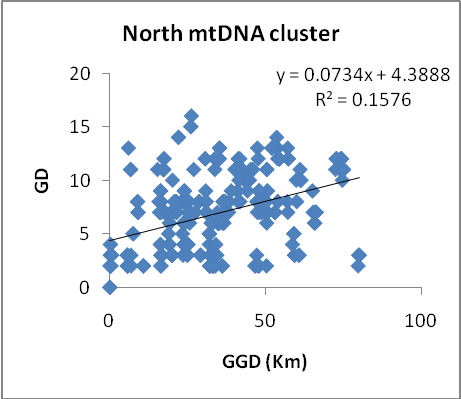

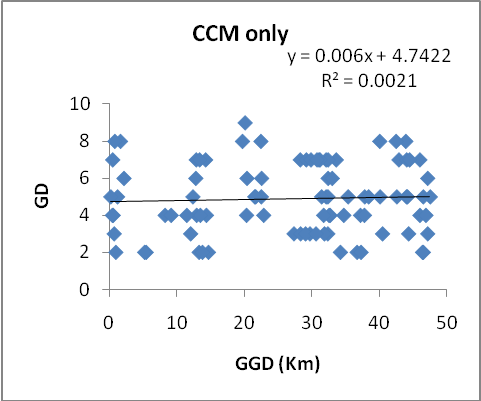


**E**

Supplement: Figure S3 — Mantel test: Genetic Distance (GD) vs Geographic Distance (GGD). Four analyses which test for possible isolation by distance were performed: A. Between the samples collected north and south of the Jezreel Valley - Rxy = 0.359, P = 0.000, R2 = 0.1292, based on 9999 permutations. B. Between the samples collected northern and southern to the Jezreel Valley excluding the CCM subspecies, Rxy = 0.412, P = 0.000, R2 = 0.1699, based on 999 permutations. C. In the samples collected southern to the Jezreel valley (in order to detect intra-cluster isolation by distance) Rxy = 0.360, P = 0.000, R2 = 0.1295, based on 9999 permutations. D. In the samples collected northern to the Jezreel Valley (same reason as C) Rxy = 0.397, P = 0.000, R2 = 0.1576, based on 9999 permutations. E. only in the CCM sub species, Rxy = 0.046, P = 0.298, R2 = 0.0021, based on 9999 permutations. (DOC) [file pone.0031372.s003.doc]

A. B. C.


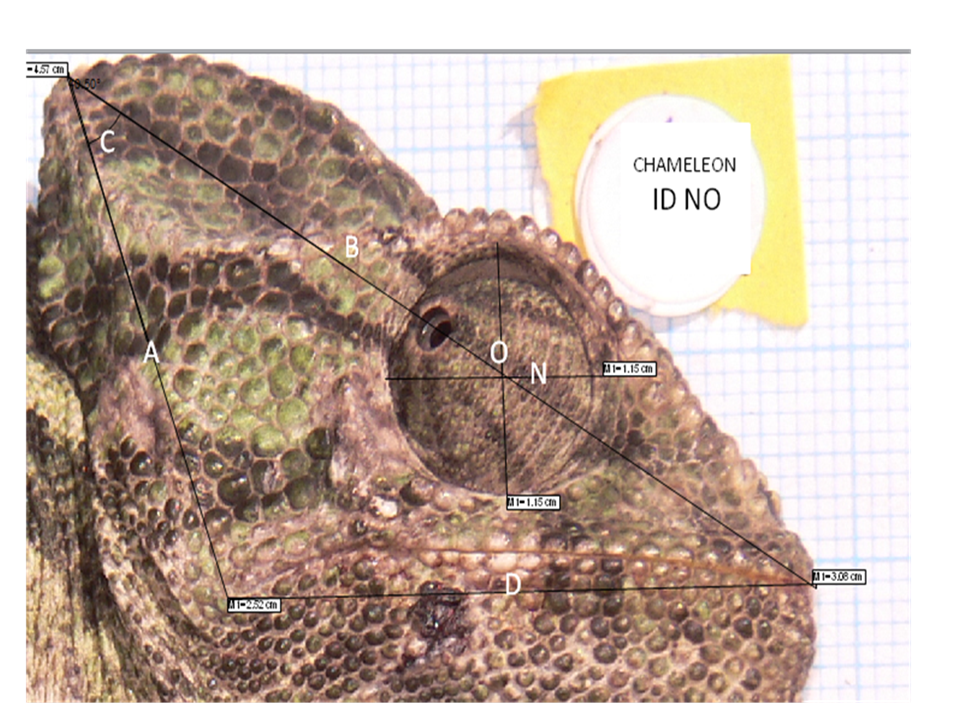

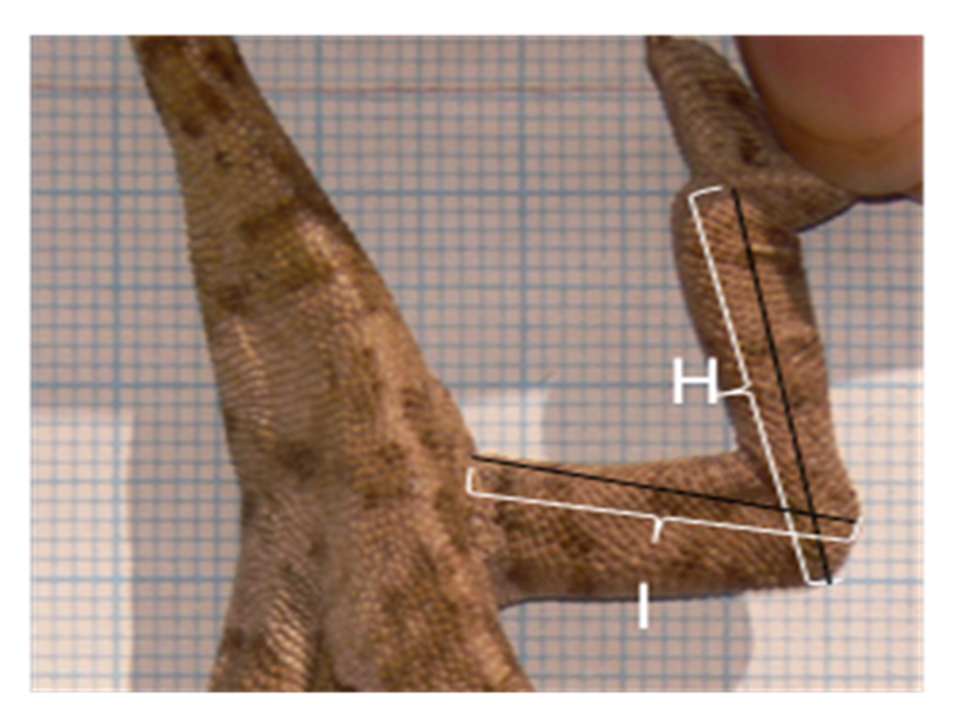


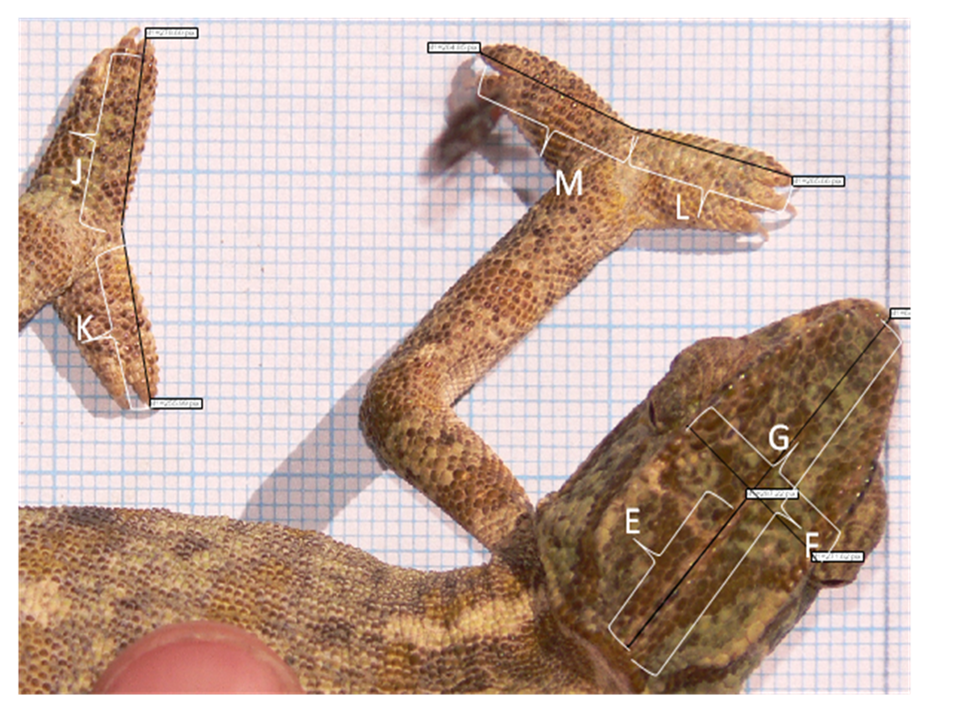
D. E. F.

Supplement: Figure S4 — Morphological measurements of the chameleons. Morphology size measurements conducted by chameleon Photography over a millimetric paper background. The chameleons were photographed (A) using a tripod (where S1 = 120 cm), Tele Macro Lens, and in bright day light for maximum aperture value (closed aperture) in order to increase the depth of field. (B) Skull measurements. (C–D) Measurements of other body parts. (E) Perspective distortion effect. The chameleon portrait was digitally measured using the background as size calibration. Perspective distortion generated by the distance X cause Figure 2 look bigger then Figure 1 although both represent objects with identical sizes. From the geometric principle of similarity of triangles one can state that :where Object 1 represents the millimetric paper background and Object 2 is the chameleon being measured. Perspective distortion effect was tested using these characteristics (with different X sizes), as shown in (F). Example: for a 3 cm wide chameleon (X = 3 cm) we would expect less then 3% size distortion, which was defined as sufficient accuracy. For detailed explanation – see Materials and Methods. (DOC) [file pone.0031372.s004.doc]

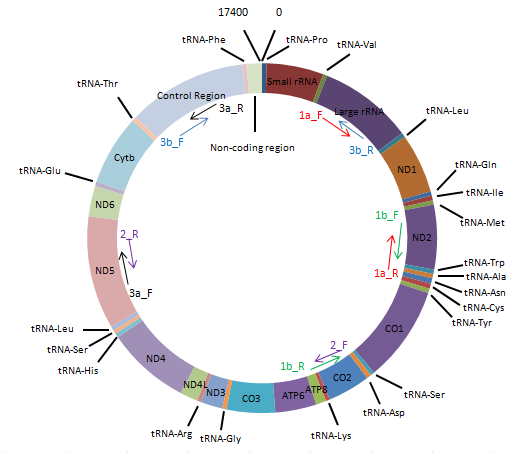

Supplement: Figure S5 — Schematic map of the mitochondrial genome of C. chamaeleon . Arrows designate primers used for PCR amplification of whole mtDNA in 5 overlapping fragments (for primers sequences and nucleotide positions – see Table 1. Genes were assigned according to the annotation within whole mtDNA sequence of a Turkish C. Chamaeleon (Genbank accession number EF222202.1). Genbank accession numbers were assigned to 57 C. chameleon mtDNA sequences: Forty six 637 bp mtDNA fragment sequences and 11 whole mtDNA sequences. Following are the accession numbers of the 46 short (637 bp) mtDNA fragments: JF317646 (31_Avshalom), JF317647 (37_Avshalom), JF317648 (38_Shivta_Junction), JF317649 (47_Secher_Stream), JF317650 (48_Secher_Stream), JF317651 (52_Beer_Aslug), JF317652 (64_Revivim), JF317653 (110_Nitzana), JF317654, (75_Carmel), JF317655 (106_Habonim), JF317656 (112_Caesarea), JF317657 (118_Nizzanim), JF317658 (129_Kishon), JF317659 (136_Mt.Gahar), JF317660 (150_Salem), JF317661 (151_Salem), JF317662 (164_Tivon), JF317663 (165_Tivon), JF317664 (71_Haon), JF317665 (77_Akbara_Stream), JF317666 (78_Akbara_Stream), JF317667 (81_Akbra_Stream), JF317668 (86_Korazim), JF317669 (88_Korazim), JF317670 (90_Korazim), JF317671 (94_Ramot), JF317672 (97_Poria), JF317673 (99_Shamir), JF317674 (101_Shamir), JF317675 (102_Shamir), JF317676 (113_Baram), JF317677 (116_Fasuta), ), JN830601 (Kmehin), JN830602 (Tivon), JN830603 (Megido_Junction), JN830604 (Shivta), JN830605 (Kfar Masaryk), JN830606 (Neve_Ziv), JN830607 (Neve_Ziv), JN830608 (Beit Shean), JN830609 (Hararit), JN830610 (Magen_Shaul), JN830611 (Magen_Shaul), JN830612 (Beit_Hashita), JN830613 (Neve Ur), JN830614 (Neve Ur). Genbank accession numbers were assigned to 11 whole mtDNAs of chameleon in Israel: JF317635 (36_AVSHALOM), JF317636 (58_RAMAT_BEKA), JF317637 (177_KMEHIN), JF317638 (195_REVIVIM), JF317639 (105_HABONIM), JF317640 (108_JERUSALEM), JF317641 (169_OOSHA), JF317642 (100_SHAMIR), JF317643 (69_HAON_EAST_OF_KINERET), JF317644 (114_BARAM) [file pone.0031372.s005.doc]
