# Supplementary material for: Mitochondrial DNA Variation, but Not Nuclear DNA, Sharply Divides Morphologically Identical Chameleons along an Ancient Geographic Barrier
Source: PLoS One. 2012 Mar 13;7(3):e31372. doi: 10.1371/journal.pone.0031372 (PMC3306244; doi:10.1371/journal.pone.0031372)
Supplement: Table S1 — The normalized principal component loadings with coefficient intervals calculated from 1000 bootstrap. Variables A,B,D,E,F,G,N,O are head traits while H,I,J,K,L,M are limbs traits. (DOC) [file pone.0031372.s006.doc]

| Variable | PC1 | CI LOWER(PC1) | CIUPPER(PC1) | PC2 | CI LOWER(PC2) | CIUPPER(PC2) |
| --- | --- | --- | --- | --- | --- | --- |
| Log A (crest head height) | 1.07 | 0.869 | 1.33 | 3.31 | 2.362 | 4.504 |
| Log B (crest–mouth length) | 0.98 | 0.787 | 1.218 | 2.53 | 1.868 | 3.367 |
| Log D (jaw length) | 1.05 | 0.856 | 1.272 | 1.143 | -0.072 | 2.033 |
| Log E (dorsal crest length) | 1.066 | 0.805 | 1.417 | 4.92 | 2.598 | 7.56 |
| Log F (dorsal crest–mouth) | 0.941 | 0.729 | 1.262 | 4.873 | 2.592 | 6.997 |
| Log G (dorsal crest width) | 0.855 | 0.619 | 1.172 | 1.61 | 0.587 | 2.752 |
| Log H (elbow-hand joint) | 1.165 | 0.898 | 1.518 | -1.116 | -2.202 | -0.233 |
| Log I (armpit-elbow) | 1.08 | 0.819 | 1.449 | -0.501 | -2.08 | 1.063 |
| Log J (digitus tertius) | 1.382 | 0.987 | 1.941 | -4.072 | -4.886 | -3.149 |
| Log K (annulus finger) | 1.392 | 0.961 | 1.991 | -4.056 | -5.144 | -2.982 |
| Log L (foot digitus tertius) | 1.351 | 0.97 | 1.859 | -3.638 | -4.295 | -2.931 |
| Log M (foot annulus finger) | 1.304 | 0.902 | 1.888 | -3.868 | -4.719 | -2.898 |
| Log N (eye vertical diameter) | 0.74 | 0.544 | 0.839 | 3.242 | 1.648 | 4.906 |
| Log O (eye horizontal diameter) | 0.667 | 0.486 | 0.767 | 3.372 | 1.753 | 5.096 |
