# Supplementary material for: Mitochondrial DNA Variation, but Not Nuclear DNA, Sharply Divides Morphologically Identical Chameleons along an Ancient Geographic Barrier
Source: PLoS One. 2012 Mar 13;7(3):e31372. doi: 10.1371/journal.pone.0031372 (PMC3306244; doi:10.1371/journal.pone.0031372)
Supplement: Table S2 — Stepwise discriminant function analysis. Six parameters of head and limbs traits define the minimal function needed to distinguish between the two subspecies. Wilks' lambda = 0.214, df = 36,582 P<0.001. The minimal canonical variant (standardized by within-group variances), required for best separating between the sub-species is: CV1 = 1.018×logB+0.256×logE+0.404×logI−1.386×logK−0.902×logM+0.379×logO. (DOC) [file pone.0031372.s007.doc]

| Parameter | F-to-remove |
| --- | --- |
| LogB(crest–mouth length) | 3.13 |
| LogE(crest length ) | 2.72 |
| logI(arm length) | 3.02 |
| logK(medial foot pad length) | 5.49 |
| logM(medial hand pad length) | 2.2 |
| LogO(eye diameter) | 4.8 |
