# Supplementary material for: Mitochondrial DNA Variation, but Not Nuclear DNA, Sharply Divides Morphologically Identical Chameleons along an Ancient Geographic Barrier
Source: PLoS One. 2012 Mar 13;7(3):e31372. doi: 10.1371/journal.pone.0031372 (PMC3306244; doi:10.1371/journal.pone.0031372)
Supplement: Table S3 — Primers list (F-Forward, R-Reverse) for whole mtDNA amplification. Nucleotide positions were assigned using the whole mtDNA sequence of a Turkish Chamaeleo Chamaeleon (Genbank accession number EF222202.1). For the content of mt genes in each fragment see Figure S5. (DOC) [file pone.0031372.s008.doc]

| Primer | Sequence | Nucleotide position |
| --- | --- | --- |
| 1a- F | AGTAGCACTACACCCAACAGACCC | 1733 -1756 |
| 1a-R | ATCAAGGCCTACTAGTCCTG | 5044 -5023 |
| 1b- F | GCTGCCCCAATTTACTTCTG | 4146 - 4165 |
| 1b- R | GTTATCACGGTCAGATTCAAGGGG | 7850 - 7827 |
| 2- F | CATGAACACTCCCAGCCTTAGGAG | 7441 -7444 |
| 2- R | TCGGTGGGTGATTTTGAAGAAGGC | 13206 -13183 |
| 3a- F | AACCGCTATAACATCCGCCTACTC | 12855- 12878 |
| 3a- R | ATCGGTCGAGAGCTCGATATGTGG | 16495- 16472 |
| 3b-F | AATGTGCTGCGGTCCTTACAAGCC | 16607-16630 |
| 3b-R | CAGTATTACTGGGTCGTGTG | 2236-2217 |
