# Supplementary material for: Mitochondrial DNA Variation, but Not Nuclear DNA, Sharply Divides Morphologically Identical Chameleons along an Ancient Geographic Barrier
Source: PLoS One. 2012 Mar 13;7(3):e31372. doi: 10.1371/journal.pone.0031372 (PMC3306244; doi:10.1371/journal.pone.0031372)
Supplement: Table S4 — List of primers used to sequence the whole chameleon mtDNA. Nucleotide positions were assigned using the whole mtDNA sequence of a Turkish Chamaeleo Chameleon (Genbank accession number EF222202.1). (DOC) [file pone.0031372.s009.doc]

| Primer | Sequence | Nucleotide position |
| --- | --- | --- |
| 1 | AGTAGCACTACACCCAACAGACCC | 1733 -1756 |
| 2 | TAGGGATAACAGCGCTACCTTCTC | 2257- 2280 |
| 3 | TCCTTCAACCTATTACAGACGGCG | 2803- 2826 |
| 4 | TCCACTAACACTAGCAGCCC | 3600-3619 |
| 5 | GCTGCCCCAATTTACTTCTG | 4146-4165 |
| 6 | AGTAGTCACATGGTTAGGTGG | 5238 -5260 |
| 7 | AAACCAGCGGGCATTAATCCAGTC | 5107- 5131 |
| 8 | TAGCATCATCTAAAGCCGGAACCG | 5586- 5609 |
| 9 | CTAGGATTCATGGTCTGAGCCCAC | 6095 -6118 |
| 10 | GACGATATTCAGACTTCCCAGACG | 6561- 6584 |
| 11 | GTTATCACGGTCAGATTCAAGGGG | 7850- 7827 |
| 12 | CATGAACACTCCCAGCCTTAGGAG | 7441-7444 |
| 13 | TCCCAGAACTATGTGGCATC | 7874- 7893 |
| 14 | AGCTGTAGCCCTAATCCAAG | 8445- 8464 |
| 15 | GGTTGTGGAGTTGATTGCTC | 9610 - 9591 |
| 16 | ACATTCTTCGTAGCCACAGG | 9100-9119 |
| 17 | ATCCTCTAGGCAATGCACGC | 9486-9505 |
| 18 | AGCAAGCACAGGACTAGCAC | 9979-9998 |
| 19 | TGGCTGTGATGGTTGCTGCTGATC | 11254-11231 |
| 20 | TGCGAATTTCACCCATGCTATGCC | 10775-10798 |
| 21 | GGATCAGCAGCAACCATCAC | 11250- 11269 |
| 22 | GACCTTAGGAGTCAAGACCC | 11567- 11587 |
| 23 | TGCAGGCAGCATGATCCAAC | 12035-12054 |
| 24 | TCGGTGGGTGATTTTGAAGAAGGC | 13206-13183 |
| 25 | AACCGCTATAACATCCGCCTACTC | 12855-12878 |
| 26 | AGGACCAAAAGCCATCACAC | 13286- 13305 |
| 27 | TTCTCACCTGGACTAAAACC | 13930-13949 |
| 28 | GGTTACATCCTACCCTGAGG | 14385- 14404 |
| 29 | GCCTACACAATCCTACGATC | 14826-14845 |
| 30 | ACTATCCAAGCTAGGCCTC | 15316- 15334 |
| 31 | GGCTGTTATCGTTAAATTGCG | 16001-15981 |
| 32 | ATCGGTCGAGAGCTCGATATGTGG | 16495- 16472 |
| 33 | ACGAAAAGTTAGGAGATCGG | 16871- 16890 |
| 34 | CGACCGTGAAAAATTCAAAACACC | 16756 -16779 |
| 35 | TTGGTCCTGGACTTGGTGTG | 17446 - 17407 |
| 36 | GCTGTTCGTTATTTGGGGTC | 348-329 |
| 37 | CATAACGCCAAGATACCCAC | 178-197 |
| 38 | ACGACAGGTCAAGGTGTAGC | 720-739 |
| 39 | AGACCAAGAAGTAGTCGGCC | 1348-1367 |
| 40 | TGCAAAGGTAGCACAATCAC | 1966-1985 |
| 41 | AACAGCTAGCTGCCCAAACC | 5063- 5082 |
| 42 | CAGCCACCAGTAAATATCGC | 1589 -1608 |
| 43 | GCCACACCAAAACTGCACTG | 788- 807 |
| 44 | CAAACCAGCCATAACAACGG | 13810- 13829 |
| 45 | GACTGATCATACGAATAGAGG | 5816 -5796 |
| 46 | GGTCTAGATGGTGTTAGAGG | 2227 - 2208 |
| 47 | CGTCTGATATGTATCCAGAG | 13725- 13706 |
| 48 | CCGGTTGGTTTAGTAGGAGAAC | 1661- 1640 |
| 49 | CTGTAGGTCGCTTTGTCTAG | 1287-1268 |
