# Supplementary material for: Mitochondrial DNA Variation, but Not Nuclear DNA, Sharply Divides Morphologically Identical Chameleons along an Ancient Geographic Barrier
Source: PLoS One. 2012 Mar 13;7(3):e31372. doi: 10.1371/journal.pone.0031372 (PMC3306244; doi:10.1371/journal.pone.0031372)
Supplement: Table S5 — mtDNA RFLP screen of C. chamaeleons in Israel. (DOC) [file pone.0031372.s010.doc]

|  | mtDNA profile of the chameleons of the Carmel and southern type | mtDNA profile of the chameleons of the northern type |
| --- | --- | --- |
| BsmAI | 894 bp | 269,625bp |
| AseI | 414,480bp | 894 bp |
